# Supplementary figures and images for: Candida albicans Isolates 529L and CHN1 Exhibit Stable Colonization of the Murine Gastrointestinal Tract
Source: mBio. 2021 Nov 2;12(6):e02878-21. doi: 10.1128/mBio.02878-21 (PMC8561340; doi:10.1128/mBio.02878-21)

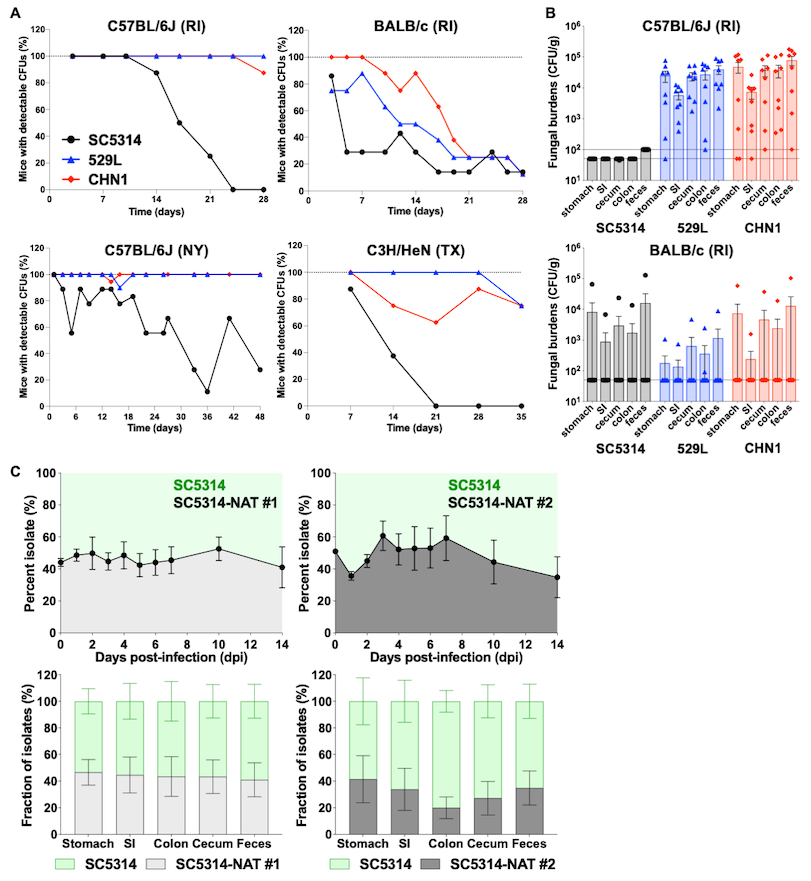

Supplement: FIG S1 [file mbio.02878-21-sf001.tif]

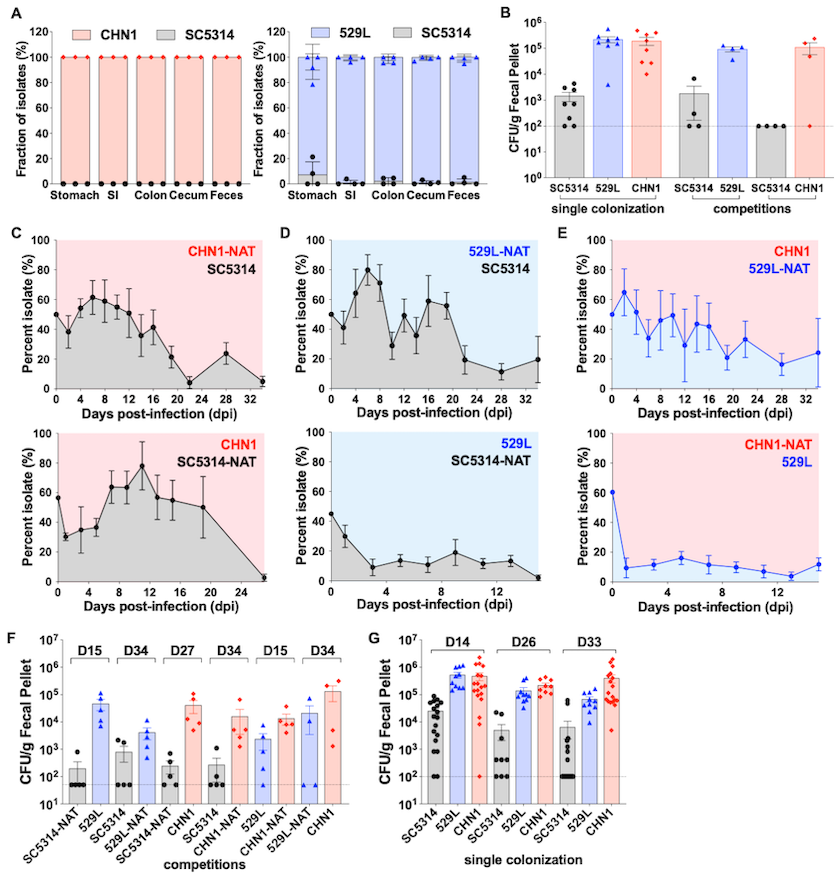

Supplement: FIG S2 [file mbio.02878-21-sf002.tif]

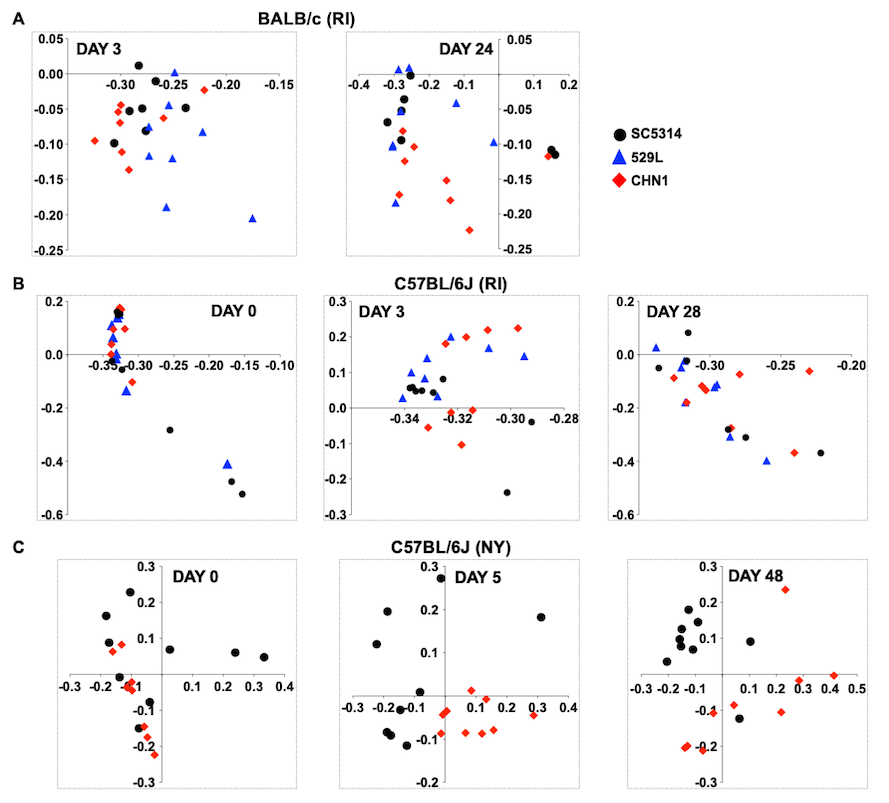

Supplement: FIG S3 [file mbio.02878-21-sf003.tif]

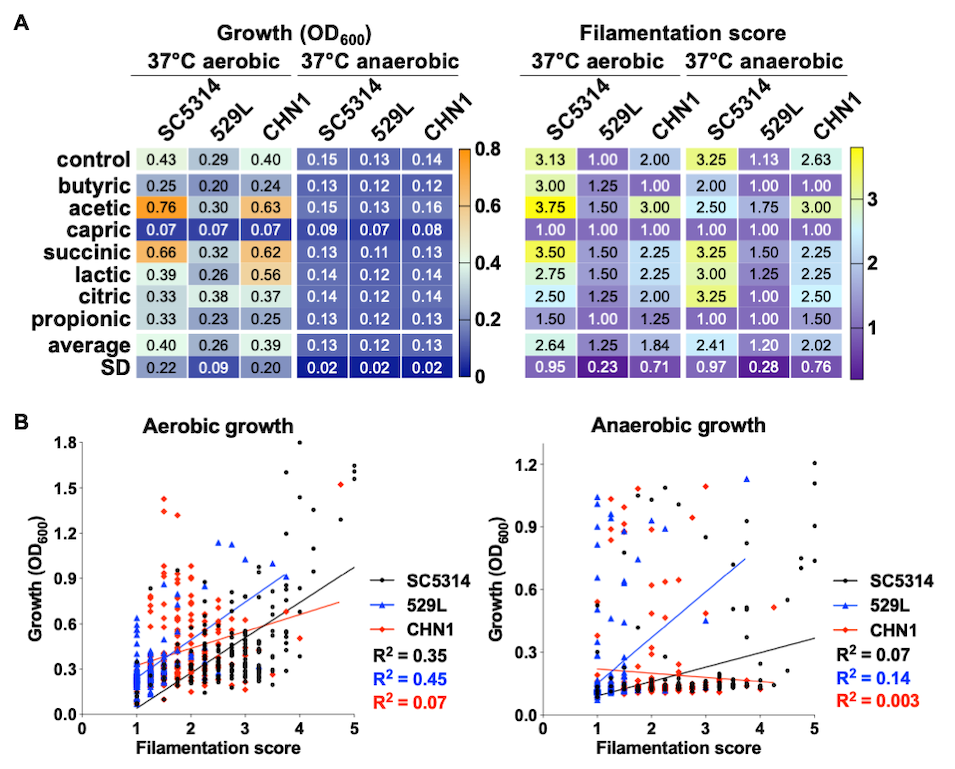

Supplement: FIG S4 [file mbio.02878-21-sf004.tif]

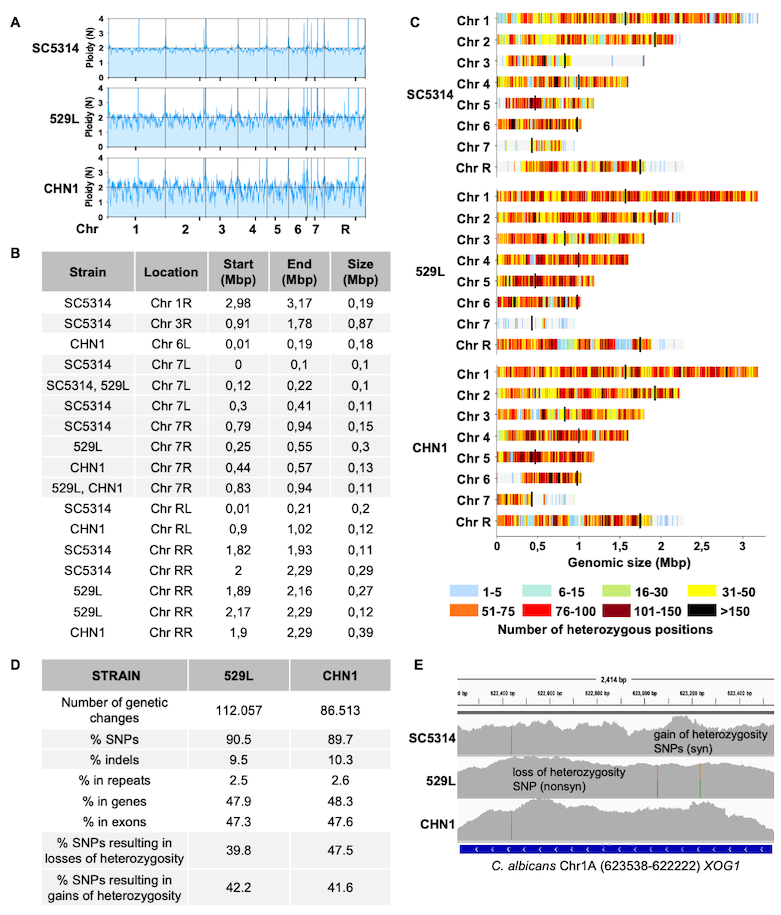

Supplement: FIG S5 [file mbio.02878-21-sf005.tif]

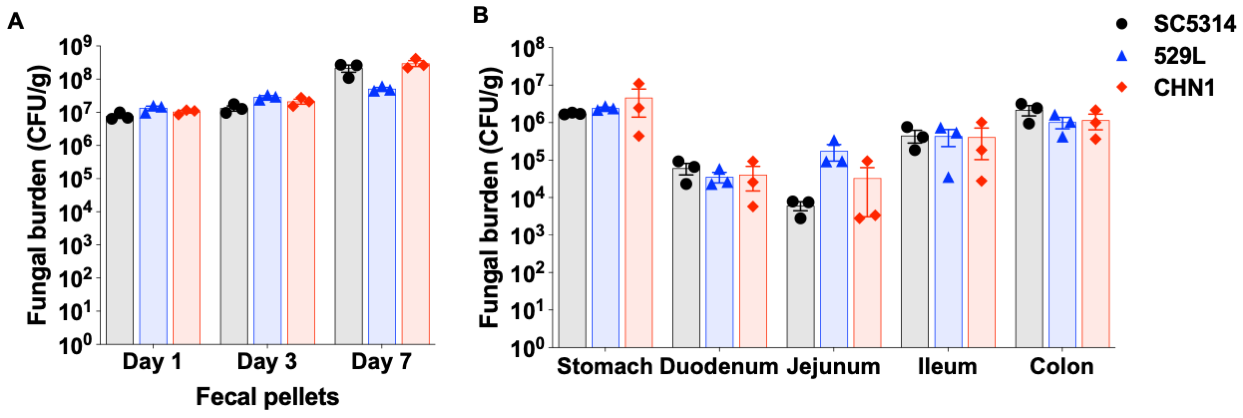

Supplement: FIG S6 [file mbio.02878-21-sf006.tif]
